# A novel gene, TARDBP, and the protein it encodes can predict glioma patient prognosis and establish a prediction model

**DOI:** 10.1186/s12883-023-03224-4

**Published:** 2023-05-06

**Authors:** Xu Fang, Fan Wu, Chen Jiang

**Affiliations:** 1 Department of Neurosurgery, the Affiliated Wuxi People’s Hospital of Nanjing Medical University, Wuxi People’s Hospital, Wuxi Medical Center, Nanjing Medical University, Wuxi, China; 2 Department of Orthopaedics and Traumatology, Shanghai East Hospital, School of Medicine, Tongji University, Shanghai, China; 3 Department of Neurosurgery Intensive Care Unit, the Affiliated Wuxi People’s Hospital of Nanjing Medical University, Wuxi People’s Hospital, Wuxi Medical Center, Nanjing Medical University, Wuxi, China

**Keywords:** TARDBP gene, Glioma patients, Survival, Prediction model

## Abstract

**Background:**

TDP-43 (43-kD transactive response DNA-binding protein) is a DNA-/RNA-binding protein that plays an important role in several nervous system diseases, such as amyotrophic lateral sclerosis (ALS) and frontotemporal dementia (FTD). Whether it plays an important role in glioma patients is unknown.

**Methods:**

Datasets were downloaded from the Chinese Glioma Genome Atlas (CGGA) website (http://www.cgga.org.cn/). Cox survival analysis was performed to determine the relationship between TARDBP gene expression and the overall survival of glioma patients. GO analyses were performed to determine the biological functions of the TARDBP gene. Finally, we used PRS type, age, grade, IDH mutation status, 1p/19q codeletion status, and expression value of the TARDBP gene to construct a prediction model. With this model, we can predict patients’ 1-, 2-, 3-, 5-, and 10-year survival rates.

**Results:**

The TARDBP gene plays an important role in glioma patients. The expression of the TARDBP gene has a significant correlation with glioma patient survival. We also constructed an ideal prediction model.

**Conclusion:**

Our findings suggest that the TARDBP gene and the protein it encodes play important roles in glioma patients. The expression of the TARDBP gene has a significant correlation with the overall survival of glioma patients.

**Supplementary Information:**

The online version contains supplementary material available at 10.1186/s12883-023-03224-4.

## Introduction

TDP-43 (43-kD transactive response DNA-binding protein), encoded by the TARDBP gene, is a DNA-/RNA-binding protein that is usually localized to the nucleus [1, 2] and plays an important role in several diseases of the nervous system, such as amyotrophic lateral sclerosis (ALS) and frontotemporal dementia (FTD) [3, 4]. TDP-43 plays a key role in RNA metabolism at multiple stages, including transcriptional regulation, pre-mRNA splicing, RNA processing, and RNA localization, in ALS, FTD, and many other neurodegenerative diseases [5]. Cytoplasmic TDP-43 inclusions are also a key feature of limbic-predominant age-related TDP-43 encephalopathy (LATE), a newly recognized neurodegenerative disorder that mimics Alzheimer’s disease [6]. Therefore, we infer that the TARDBP gene and mutations thereof also play an important role in several neurological diseases. Glioma is the most prevalent type of brain tumour and has the highest fatality rate in adults [7]. In most cases, the symptoms and signs reported the year before diagnosis are nonspecific. Characteristic modes of clinical presentation include headache, fatigue, new-onset epilepsy, focal deficits (such as pareses or sensory disturbances), neurocognitive impairment, and symptoms and signs of increased intracranial pressure [8]. These tumours account for 70% of adult malignant primary brain tumours. The annual incidence of gliomas is approximately six cases per 100,000 individuals worldwide. Men are 1.6-fold more likely to be diagnosed with gliomas than women [9]. Conventional therapies include surgery, radiotherapy, and pharmacotherapy (typically chemotherapy with temozolomide). Glioma patients consistently have a poor prognosis. Their median survival is less than 2 years with the current standard of care of maximal safe resection, followed by combined radiochemotherapy with the alkylating agent temozolomide [10]. However, research reports focused on the role of the TARDBP gene in the occurrence, development and prognosis of glioma are rare. Using the Chinese Glioma Genome Atlas (CGGA) database, we analysed the TARDBP gene expression profile in various patients and explored whether the expression of the TARDBP gene was associated with overall survival.

## Methods

### Patients and cohort inclusion

In total, 1018 patients with follow-up information and gene expression information were obtained from the CGGA website (http://www.cgga.org.cn/). The training group had a total of 693 patients [11–13], and the validation group had 325 patients [11, 14, 15]. According to the CGGA website, both the training and validation groups data types were subjected to mRNA sequencing; however, the platform of the training group was Illumina HiSeq, and the platform of the validation group was Illumina HiSeq 2000 or 2500. For all patients, several types of clinical information were available, such as WHO grade [16], sex, overall survival (OS), and censoring, and a total of 23,961 different gene expression levels were determined.

### Cox survival analysis of the relationship between TARDBP gene expression and overall survival

We analysed the relationships between TARDBP gene expression or other characteristics, such as age, sex, and IDH mutation, and glioma patient OS. The end point of Cox survival analysis was determined by censoring. Based on the positive factors from the univariate analysis, multivariate analyses were performed.

### Functional enrichment analysis

We obtained the most relevant gene list of the TARDBP gene by Pearson correlation analysis. The gene list was arranged in descending order, and the top 500 genes were selected and uploaded to The Database for Annotation, Visualization and Integrated Discovery (DAVID, https://david.ncifcrf.gov/. Version 6.8). The official gene symbol and Homo sapiens were chosen as analysis parameters. Finally, Gene Ontology (GO) analysis included GOTERM BP DIRECT (BP), GOTERM CC DIRECT (CC), and GOTERM MF DIRECT (MF), and Kyoto Encyclopedia of Genes and Genomes (KEGG) pathway [17–19] analysis enrichment results were downloaded. The top six results in ascending order of the *P* value are displayed in this study.

### Biological functional enrichment

First, from the results of GO and KEGG analyses, we selected the 20 genes with the lowest *P* values. Second, we analysed the correlation of the genes in the GO and KEGG gene enrichment lists and the TARDBP gene by Pearson correlation analysis. The results were arranged in descending order of the R-value.

### Prognostic difference analysis

We divided patients in both the training and validation groups into 2 groups according to the TARDBP gene expression value. The grouping criterion was the average expression value of the TARDBP gene. Individuals with values higher than the average were placed in the higher group, and those with values lower than the average were placed in the lower group (the average value of the training group was 70, and the average value of the validation group was 69). Kaplan‒Meier curves (K‒M curves) were drawn to display the prognostic differences. The significance of the prognostic value was tested by a log-rank test. *P* < 0.05 was considered statistically significant. The K‒M curves were drawn in R.

### Construction of the predictive overall survival rate model

Six factors, including PRS type, age, grade, IDH mutation status, 1p/19q codeletion status, and expression value of the TARDBP gene, were used to construct the prediction model. The 1-, 2-, 3-, 5-, and 10-year survival rates of patients could be predicted by the total points and sum points of every factor. Calibrated curves and C-index values were used to display the accuracy of the prediction model.

### Statistical analysis and plots

Cox survival analysis was performed using IBM SPSS Statistics software (Version 19, Chicago, IL). GSVA analyses were implemented with the GSVA package in the R environment. Gene Ontology (GO) enrichment analysis and Kyoto Encyclopedia of Genes and Genomes (KEGG) pathway enrichment analysis were performed in The Database for Annotation, Visualization and Integrated Discovery (DAVID, https://david.ncifcrf.gov/, Version 6.8). The general GO and KEGG gene list was downloaded from the website (http://download.baderlab.org/EM_Genesets/current_release/Human/symbol). For all statistical methods, *P* < 0.05 was considered significant. The heatmap, violin plot, scatter diagram and calibration plot were all made in R (https://www.r-project.org/ Version 4.2.1).

## Results

### Expression of the TARDBP gene is correlated with the prognosis of glioma patients

**Fig. 1:**
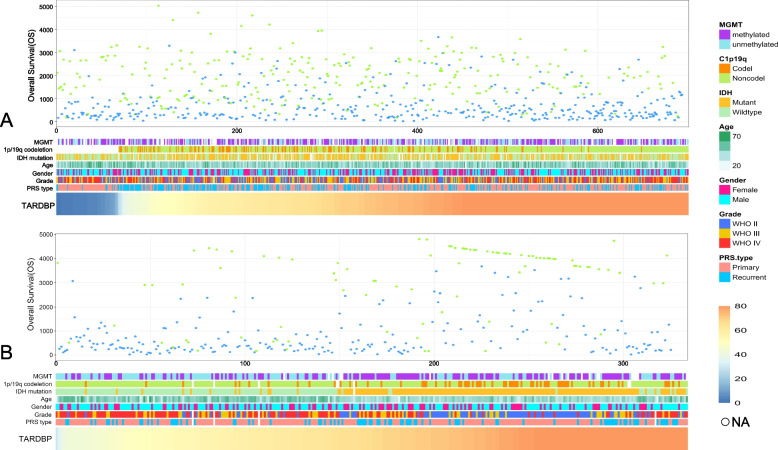
The relationship between the expression value of the TARDBP gene and clinical characteristics. **A** is the training group. **B** is the validation group. All data are arranged in ascending order of the expression value of the TARDBP gene

**Fig. 2:**
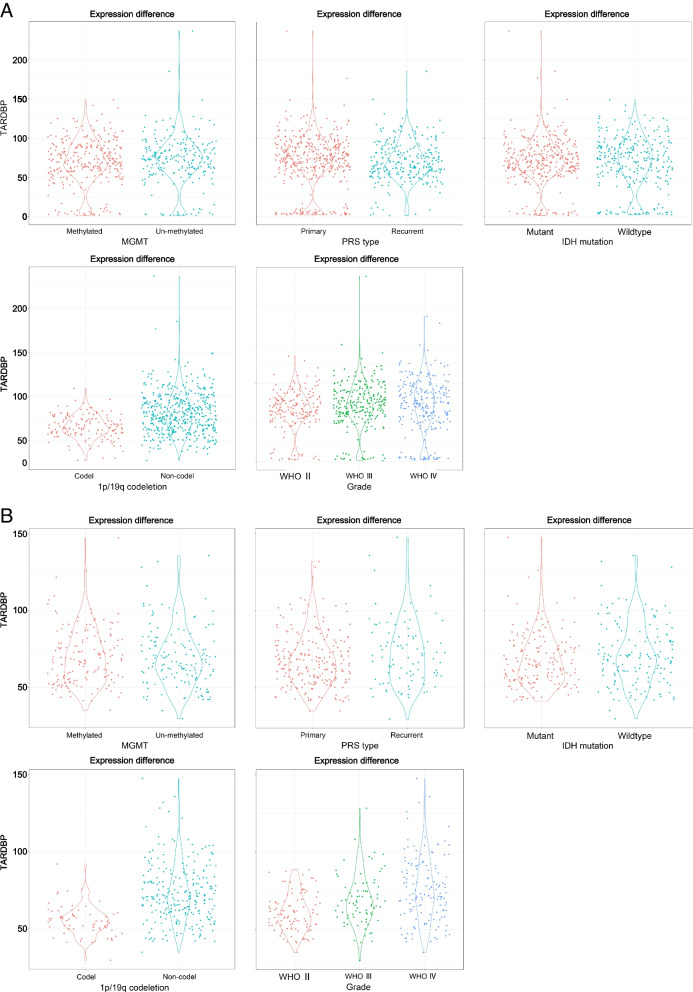
The expression value of the TARDBP gene with respect to various factors. **A** is the training group. **B** is the validation group. All differences were tested by Student’s t test, and *P* < 0.05 was considered statistically significant. (*P < 0.05; **P < 0.005; ***P < 0.001)

A total of 1,018 patients were enrolled in this study, and their information is displayed in Fig. 1. All patients were arranged in ascending order of the TARDBP gene expression value. In the training group, the expression of the TARDBP gene was significantly different between the 1p/19q codeletion groups (*p* < 0.001) (Fig. 2). Additionally, the expression of the TARDBP gene was significantly different between WHO grade II and WHO grade III (*p* = 0.03) (Fig. 2), but there was no difference between WHO grade II and WHO grade IV (*p* = 0.067) (Fig. 2) or between WHO grade III and WHO grade IV (*p* > 0.05) (Fig. 2). Moreover, the expression of the TARDBP gene showed no statistically significant difference according to MGMT status, PRS type or IDH mutation (*p* > 0.05) (Fig. 2). In the validation group, the expression of the TARDBP gene showed statistically significant differences according to 1p/19q codeletion status (*p* < 0.001) (Fig. 2), between WHO grade II and WHO grade III (*p* < 0.001) (Fig. 2), between WHO grade II and WHO grade IV (*p* < 0.001) (Fig. 2), between WHO grade III and WHO grade IV (*p* < 0.01) and according to PRS type (*p* = 0.024) (Fig. 2).

We then analysed whether various factors were significantly correlated with patient survival. We found that in the training group, expression of the TARDBP gene, PRS type, age, IDH mutation status, 1p/19q codeletion status, WHO grade and MGMT status significantly correlated with glioma patients’ OS. Radiation therapy, temozolomide (TMZ) treatment and sex had no correlation with glioma patient OS. Therefore, expression of the TARDBP gene, PRS type, age, IDH mutation status, 1p/19q codeletion status, WHO grade and MGMT status were included in the Cox proportional hazards model (Cox model). We found that the expression level of the TARDBP gene is an independent predictor of the OS of glioma patients. In the validation group, the expression level of the TARDBP gene was also an independent predictor of glioma patient OS (Table 1).

**Table 1:** Univariate and multivariate survival analysis results of various factors in the training group and validation group

	Univariate analysis		Multivariate analysis	
	Exp(B)(95%CI)	P	Exp(B)(95%CI)	P
**Univariate and multivariate prognostic analysis in the training group overall survival (OS)**				
TARDBP	1.005(1.001-1.008)	0.009	1.006(1.001-1.011)	0.019
PRS type	2.182(1.785-2.667)	<0.001	2.515(1.956-3.234)	<0.001
Age	1.026(1.018-1.035)	<0.001	1.013(1.004-1.023)	0.006
IDH mutation	0.323(0.262-0.399)	<0.001	0.588(0.431-0.804)	<0.001
1p/19q codeletion	0.268(0.193-0.372)	<0.001	0.447(0.295-0.678)	<0.001
Grade	2.545(1.846-3.508)	<0.001	2.247(1.489-3.391)	<0.001
Grade*	6.968(5.081-9.554)	<0.001	3.814(2.451-5.937)	<0.001
Radiation therapy	1.241(0.953-1.615)	0.109	-	-
TMZ treatment	1.242(0.974-1.586)	0.081	-	-
MGMT methylation	0.795(0.639-0.990)	0.041	0.918(0.72-1.17)	0.489
Gender	1.061(0.868-1.296)	0.564	-	-
**Univariate and multivariate prognostic analysis in the validation group overall survival (OS)**				
TARDBP	1.028(1.021-1.034)	<0.001	1.017(1.009-1.025)	<0.001
PRS type	2.874(2.160-3.824)	<0.001	3.095(2.177-4.400)	<0.001
Age	1.033(1.020-1.046)	<0.001	1.015(1.001-1.029)	0.030
IDH mutation	0.355(0.269-0.468)	<0.001	0.645(0.447-0.929)	0.018
1p/19q codeletion	0.170(0.104-0.277)	<0.001	0.349(0.201-0.605)	<0.001
Grade	3.497(2.287-5.348)	<0.001	2.900(1.806-4.655)	<0.001
Grade*	8.896(5.992-13.206)	<0.001	5.144(3.152-8.396)	<0.001
Radiation therapy	0.632(0.457-0.872)	0.005	0.945(0.649-1.376)	0.768
TMZ treatment	1.445(1.078-1.937)	0.014	0.508(0.360-0.718)	<0.001
MGMT methylation	0.830(0.632-1.089)	0.178	-	-
Gender	0.941(0.716-1.236)	0.660	-	-

### The TARDBP gene regulates mRNA splicing and RNA binding

**Fig. 3:**
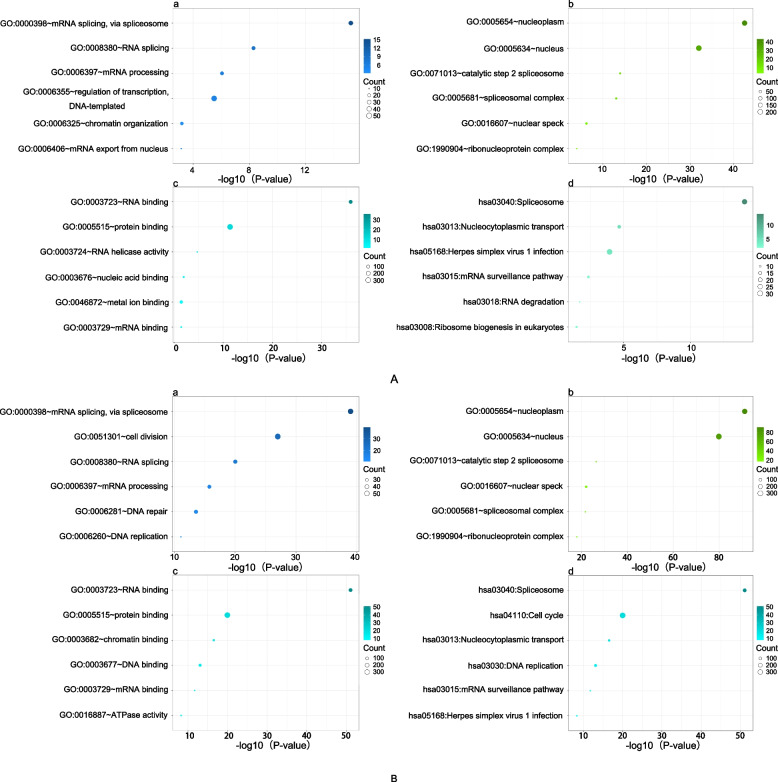
The results of biological functional enrichment analysis of the TARDBP gene on the DAVID website. **A** is the training group. **B** is the validation group. **a** BP, **b** CC, **c** MF and **d** KEGG

To determine the function of the TARDBP gene in glioma patients, we selected the top 500 correlated genes from Pearson correlation analysis (R > 0.5, descending arrangement, *p* < 0.05) and conducted DAVID analysis. The results of the GO and KEGG analyses described above were saved. The top 6 GO and KEGG analysis results were arranged in ascending order by *p* value, as displayed in Fig. 3. In terms of biological processes, we found that the TARDBP gene mainly participates in mRNA splicing, RNA splicing and mRNA processing. In terms of cellular components, the TARDBP gene is mainly distributed in internal compartments, such as the nucleoplasm and nucleus. In terms of molecular functions, the TARDBP gene mainly participates in RNA, protein and mRNA binding. Moreover, the TARDBP gene mainly functions in spliceosome-related signalling. The validation group had similar results, supporting our findings (Fig. 3).

### GSVA determined the function of the TARDBP gene

**Fig. 4:**
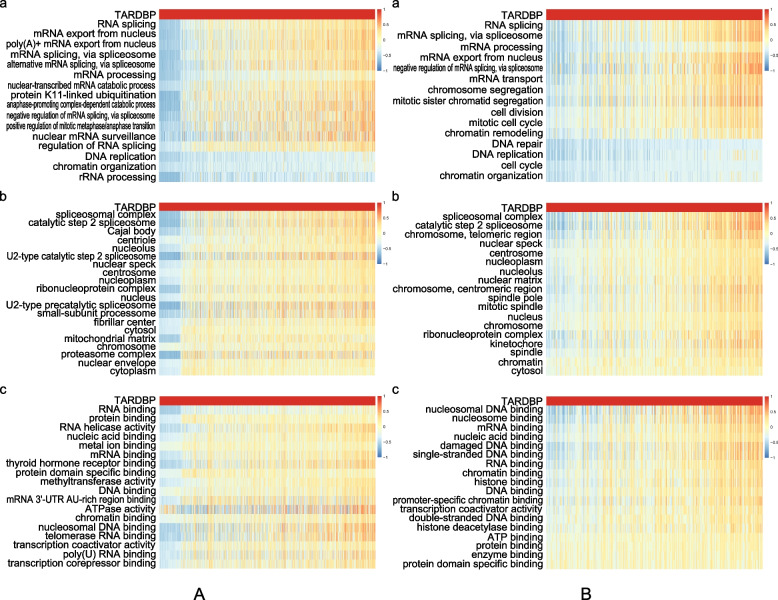
The GSVA results of TARDBP biological functional enrichment analysis. **A** is the training group. **B** is the validation group. All items are arranged in descending order of the R value. The R values were calculated using the Pearson correlation analysis method in the R environment

To further determine the function of the TARDBP gene, we performed GSVA (Gene Set Variation Analysis). We selected genes with the same functions in terms of BP, CC and MF. Between the training and validation groups, we confirmed that the TARDBP gene participates in RNA splicing and exists in the spliceosomal complex. In terms of MF, the results of the two groups showed differences. KEGG enrichment did not yield enough signal pathway results, so we did not perform GSVA (Fig. 4).

### TARDBP can predict the prognosis of glioma patients’ overall survival

**Fig. 5:**
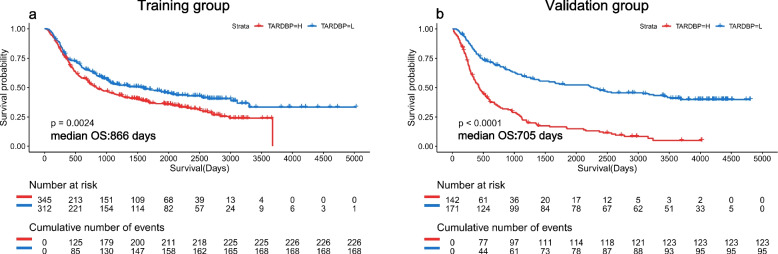
Kaplan‒Meier curves (K‒M curves) of the expression value of the TARDBP gene in each group. **a** Training group. **b** Validation group. All NA values were deleted

To determine whether the TARDBP gene can predict glioma patient prognosis, we conducted Kaplan‒Meier and Cox proportional hazard model analyses of the training and validation groups. Patients with lower expression of the TARDBP gene had longer overall survival. The validation group showed the same results (Fig. 5).

### The prediction model accurately predicts the overall survival rate of glioma patients

**Fig. 6:**
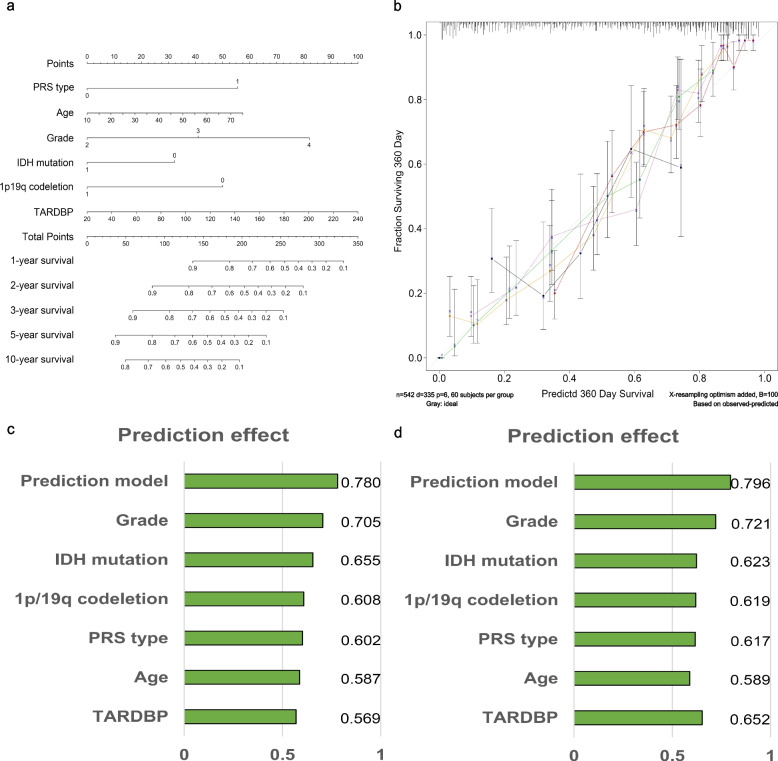
**a** The prediction model of the training group. **b** The calibration curve. **c** The C-index of the training group prediction model. **d** The C-index of the validation group prediction model

To further evaluate the predictive ability of TARDBP gene expression, we constructed a prediction model to predict the OS of glioma patients. This prediction model was based on the independent predictive factors utilized for multivariate Cox survival analyses, including PRS type, WHO grade, IDH mutation status, 1p/19q codeletion status and expression of the TARDBP gene. In this prediction model, the 1-, 2-, 3-, 5- and 10-year overall survival rates of glioma patients could be predicted. According to our model, we can calculate separate factor points of glioma patients and then obtain total points. Using this total point, we can infer the 1-, 2-, 3-, 5- and 10-year overall survival rates of patients. The calibration curve and the C-index show that our prediction model has a convincing predictive ability. The C-index of our training group prediction model is 0.78, which is higher than that of the other single factors. The C-index of our validation group prediction model was 0.796, which was also higher than that of the other single factors (Fig. 6). We also constructed the prediction model in the validation group. The prediction model and calibration curve of the validation group are shown in Supplementary Materials Fig. 1S.

## Discussion

Patients with human gliomas tend to have a poor prognosis, especially those with high-grade gliomas, which leads to different overall survival. The median overall survival is approximately 16 to 18 months in glioblastoma patients, 2 to 5 years in patients with anaplastic astrocytomas, and approximately 15 years in anaplastic oligodendroglioma patients [20]. The question of how to accurately predict the postoperative survival of glioma patients has important significance in guiding follow-up treatments. The TARDBP gene and its expression product TDP43 play an important role in a variety of brain diseases, including ASL and FTD [3, 4, 6, 21]. TDP43 is involved in RNA metabolism at multiple stages, including transcriptional regulation, pre-mRNA splicing, RNA processing, and RNA localization in ASL and FTD [5]. However, the relationship between the TARDBP gene and its expression products and patients with glioma remains to be further explored. Taking advantage of the convenience and specificity of the CGGA database, which collects and organizes a large amount of data, we aimed to illustrate the relationship between the expression of the TARDBP gene and patients with glioma. We recruited 693 patients into our training group and 325 patients into our validation group. We first analysed whether various factors, such as age, expression of the TARDBP gene, radiotherapy [22, 23], and TMZ chemotherapy [22, 23], were related to the survival prognosis of glioma patients. In the training group, we found that in the univariate analysis, TARDBP expression, PRS type, age, IDH mutation status, 1p19q codeletion status, WHO grade and MGMT status were associated with the overall survival of glioma patients, while radiation therapy, TMZ chemotherapy and sex were not related. However, this was inconsistent with the validation group, in which radiation therapy and TMZ chemotherapy were associated with patient survival prognosis, while MGMT status was not associated. We believe that this inconsistency may be due to differences between the two sets of samples. Incorporating many of the above factors related to patient prognosis into the multivariate survival analysis, we found that the expression of the TARDBP gene was also associated with the survival prognosis of patients. The validation group results also validated this finding. Therefore, we believe that the expression of the TARDBP gene is an independent factor that can predict overall survival. However, in many other studies related to gliomas, MGMT status has also been considered a key indicator of patient prognosis [24, 25]. This difference will need to be confirmed by further research.

After confirming that the expression of the TARDBP gene is closely related to the survival prognosis of patients, we aimed to gain insight into the role of the TARDBP gene and its expression products in the development of tumours in glioma patients. We first analysed the relationship between the expression of the TARDBP gene and the expression of other genes using Pearson correlation analysis. The results were arranged in descending order of their R values, and the first 500 genes were used for analysis in DAVID. The analysis results show that the TARDBP gene and its products are mainly located intracellularly, such as in nuclear plasma and nuclei, and involved in processes such as RNA splicing and mRNA processing. TDP-43, a product encoded by the TARDBP gene, is mainly involved in the binding of RNA and proteins in the occurrence and development of disease in patients with glioma. This is similar to the role that TDP-43 plays in the development of other brain diseases [26], and the specific functions and mechanisms need to be further studied.

Second, we found that high expression of the TARDBP gene indicates a poor clinical prognosis for patients. Increased expression of the TARDBP gene is accompanied by shortened patient OS. However, this finding is inconsistent with other studies. Kim et al. found that neuroblastoma patients who have lower expression levels of the TARDBP gene have a worse prognosis [27]. They found that higher TDP-43 mRNA expression was linked to a higher relapse-free survival probability. In our opinion, there are two reasons for these conflicting results. One is that the standard of measurement differs between the two studies. In our study, we chose OS as our standard. In contrast, Kim et al. chose relapse-free survival [27]. Relapse-free survival is apparently different from OS. It is possible that higher expression of the TARDBP gene can reduce relapse in postsurgical glioma patients. Another reason might be differences in the groups studied. Why the opposite conclusion was reached remains to be further studied.

Knowing that the expression of the TARDBP gene correlates with the overall survival (OS) rate of the patient, we attempted to build a model that predicts the survival rate of glioma patients. We employed six factors (expression of the TARDBP gene, age, PRS type, WHO grade, IDH mutation status, and 1p/19q codeletion status) that were independent predictors in both sets of data in the model construction. IDH mutations are associated with a favourable prognosis in glioma patients, especially in high-grade glioma patients [20, 28–31]. The 1p/19q codeletion is associated with an improved response to therapy and survival in high-grade gliomas [20, 32, 33]. The C-index of our predictive model was 0.78, which seems to be an ideal result, demonstrating that our predictive model can accurately estimate a patient's OS. For our validation group, we also used the six factors of TARDBP gene expression, age, PRS type, WHO grade, IDH mutation status, and 1p/19q codeletion status to build a predictive model. The C-index of the validation group predictive model was 0.796, which was slightly higher than that of our training group, also showing that the model we built could accurately predict a patient's OS. The results of the two groups are highly consistent, and we have reason to believe that the predictive model we constructed will be effective in predicting the prognosis of patients with glioma. MGMT status is another important factor that is correlated with improved survival and response to TMZ [34]. MGMT status could not be taken into account in the construction of our prediction model, and further research is needed. One limitation of this study is that due to its small sample size, the proportion of various predictors was inconsistent, and the standards for building prediction models were not unified. The development of more accurate predictive models requires further large-scale clinical studies. Another limitation is that additional experiments are needed to confirm our findings. Additionally, in many studies, radiotherapy and TMZ chemotherapy are the current standard of treatment. Why radiotherapy and chemotherapy have not shown importance in our research needs further study. Moreover, the reason for glioma occurrence remains unclear, and the most reliable reasons are gene mutation and ionizing radiation [35]. In glioma patients, we infer that TDP43 affects protein transmission, which plays the same role in neurodegenerative disease [36], and the specific mechanism requires further study.

In summary, we found that the TARDBP gene in glioma patients is mainly located in intracellular compartments, such as in the nuclear plasma and nucleus, and involved in processes such as RNA splicing and mRNA processing. TDP-43, a product encoded by the TARDBP gene, is mainly involved in the binding of RNA and proteins. Moreover, the expression of the TARDBP gene has a significant correlation with patient OS, and high expression of the TARDBP gene indicates poor clinical prognosis. Finally, we built a predictive model that can accurately predict patient OS.

## Supplementary Information

**Additional file 1: Fig. 1S.** (A) is the prediction model of the validation group, and (B) is the calibration curve of the validation group.

## Data Availability

The data used to support the findings of our study are available from the corresponding author upon request, and the corresponding e-mail address is 289395001@qq.com.
