# Supplementary material for: A novel gene, TARDBP, and the protein it encodes can predict glioma patient prognosis and establish a prediction model
Source: BMC Neurol. 2023 May 6;23:182. doi: 10.1186/s12883-023-03224-4 (PMC10163712; doi:10.1186/s12883-023-03224-4)
Supplement: Supplementary file 1 — Additional file 1: Fig. 1S. (A) is the prediction model of the validation group, and (B) is the calibration curve of the validation group. [file 12883_2023_3224_MOESM1_ESM.pdf]

Supplementary Materials:

Figure 1

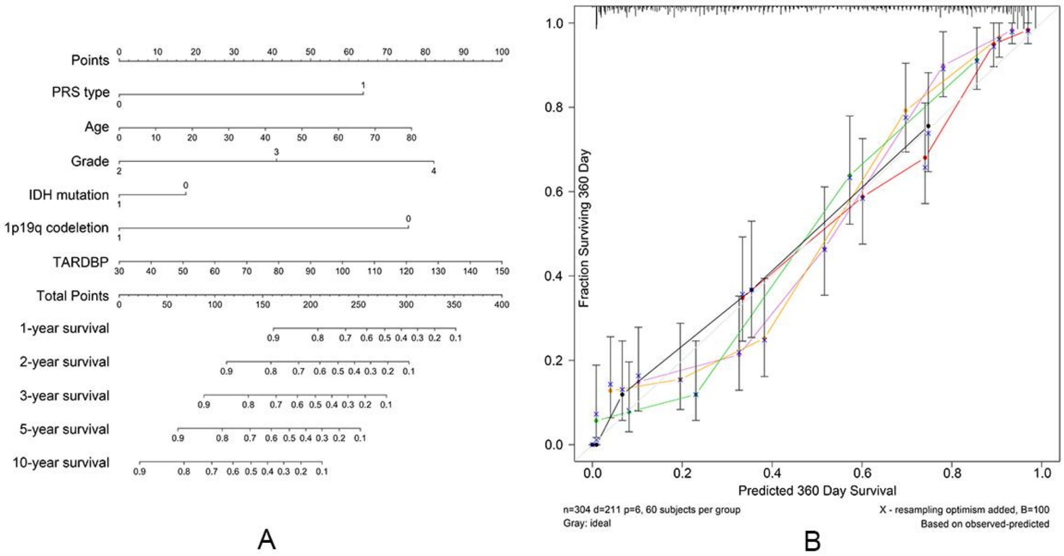

A B

Fig. 1S:(A) is the prediction model of the validation group, and (B) is the calibration curve of the validation group.
